# A Rare Case of Herpes Simplex Virus-2 Hepatitis with Negative Serology

**DOI:** 10.1155/2019/4808143

**Published:** 2019-06-04

**Authors:** Mohanad Soliman, Olalekan Akanbi, Ihab Almagdub, Kishore Karri, Pradeep Yarra, Saad Emhmed Ali, Hanine El Haddad

**Affiliations:** ^1^Department of Medicine, Division of Hospital Medicine, University of Kentucky, Lexington, KY, USA; ^2^Department of Medicine, Division of Infectious Diseases, University of Kentucky, Lexington, KY, USA

## Abstract

Herpes simplex virus-2 (HSV2) hepatitis represents a rare but serious complication of HSV2 infection that can progress to acute liver failure (ALF). We describe a case of a pregnant teenager who presented with four days of fever, headache, malaise, nausea, and vomiting. She was initially misdiagnosed with sepsis of unclear source and treated with broad-spectrum antibiotics. Empiric acyclovir was started one week into her hospitalization despite negative serologies while awaiting HSV2 PCR leading to complete resolution of symptoms. Given its high mortality and nonspecific presentation, clinicians should consider HSV hepatitis in all patients with acute hepatitis especially in high-risk population.

## 1. Introduction

We report a case of disseminated HSV-2 infection presenting as an acute liver failure (ALF) with negative serum herpes simplex virus (HSV) serology immunoglobulin M (IgM) and immunoglobin G (IgG) and without genital mucocutaneous involvement in a pregnant female during her third trimester. Despite negative serology, antiviral therapy was empirically started, and she was recovering before receiving the results of polymerase chain reaction detecting and quantifying serum level of herpes simplex 2 HSV-2.

## 2. Case Presentation

A 19-year-old female with no significant past medical history was referred to the emergency department from her antenatal clinic for further evaluation of fever 102 F, headache, malaise, nausea, and vomiting of 4-days duration following mild sore throat. She was in her 28th week of gestation on presentation. Physical examination showed an obese and anxious-appearing lady in mild respiratory distress with gravid abdomen. She weighed 142 kilograms (kg) with a BMI of 44.3. Her blood pressure was 111/60 mmHg, respiratory rate 24, heart rate 111, temperature 101.8 F, and oxygen saturation 96% on 2 liters (L) nasal cannula oxygen. She had dry mucous membranes, with no oral thrush or ulcers. Her abdomen was gravid with focal areas of tenderness, bowel sounds were heard, and fetal movement was detected. Pulmonary and cardiovascular examinations were unremarkable.

**Figure 1:**
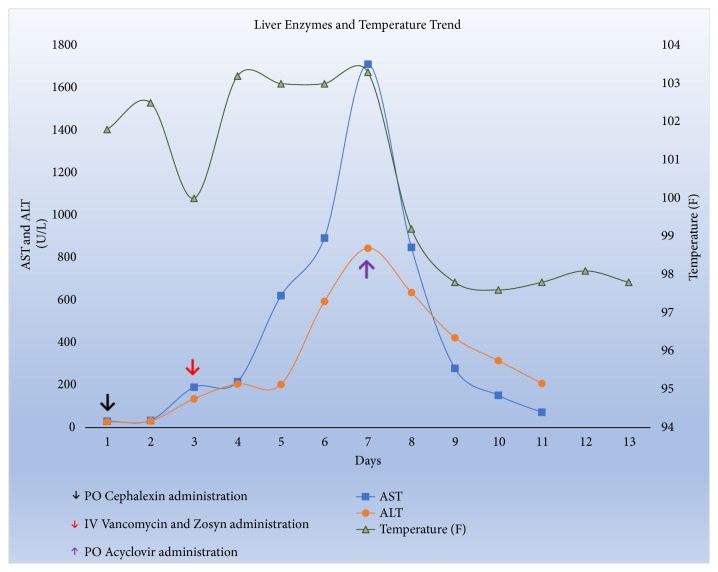
Liver enzymes and temperature trend.

Her initial laboratory tests were significant for white blood cell (WBC) count of 11.8 K/uL with 78% neutrophil predominance and lymphopenia (0.77 K/uL). Although she had no urinary symptoms, urinalysis showed bacteriuria, confirmed as group B streptococcus agalactiae (GBS) with cultures. Initial chest X-ray and liver enzymes on admission were unremarkable. She was started on intravenous fluids for suspected gastroenteritis and oral cephalexin for asymptomatic GBS infection. Stool studies for clostridium difficile, comprehensive panel, ova, and parasites were ordered which all returned negative. However, she continued to spike fever through the third day of hospitalization prompting expansion of antibiotic coverage to Vancomycin and Zosyn (Figure 1). Liver transaminases which had been normal began to spike. Aspartate aminotransferase (AST) was 190, alanine aminotransferase (AST) was 135, and alkaline phosphatase was 110 while bilirubin remained normal. Serum Tylenol and hepatitis A, B, and C serologies were negative. Infectious Disease (ID) consultation was requested who recommended further infectious work-up with CMV, EBV, HIV, HSV, Rickettsia, and Leptospira serologies.

The patient continued to spike high grade fever reaching 103 F on day 4 of admission despite being on broad-spectrum antibiotics and negative infectious work-up including the aforementioned serologies. She also developed bloody diarrhea and acute kidney injury (AKI) prompting evaluation for possible Hemolytic Uremic Syndrome (HUS) and autoimmune diseases which all returned negative as well. Right upper quadrant ultrasound showed normal liver and gallbladder morphologies. Magnetic resonance imaging of the abdomen and pelvis without contrast showed diffuse T2 hyperintensity throughout the liver compatible with acute liver injury but was negative for any focal intra-abdominal or pelvic abscess.

One week into her hospitalization, her AST and ALT kept trending up reaching a peak of 1712 U/L and 845 U/L, respectively (Figure 1), mildly elevated alkaline phosphatase 140, INR 1.6, normal total bilirubin, absolute lymphocyte count of 0.11 K/uL, persistently negative blood cultures, and serum creatinine increased to 2.9 mg/dL, all concerning for a disseminated process. She became increasingly lethargic, tachycardic, and tachypneic necessitating care in the intensive care unit. With uprising liver enzymes and negative extensive infectious and rheumatologic work-up including Hep A, B, C serologies and PCR, EBV, CMV, Parvovirus, Rickettsia, and Leptospiral serologies, as well as negative HSV 1 and 2 IgM and IgG antibodies, antinuclear, and anti-liver-kidney microsomal antibodies, normal cryoglobulins, ceruloplasmin, alpha 1 antitrypsin and serum copper levels, and largely unremarkable imaging and stool studies, a decision was made to proceed with a liver biopsy. At this time, empiric acyclovir was started while other antibiotics were discontinued per ID recommendations.

Within the first 24 hours of starting acyclovir, AST and ALT began to downtrend while fever and diarrhea improved (Figure 1). Despite negative HSV-2 serologies, the PCR was reported on hospital day-8 with a very high viral load >100 million DNA copies/ml following which she reported a remote history of cold sores but denied a history of genital ulcers. A diagnosis of HSV hepatitis was made and she was transitioned to Val-acyclovir 1 gram three times daily to complete 14 days of therapy, then to resume acyclovir 400 mg three times daily until delivery to prevent recurrence. On the 12th day of hospitalization, she was discharged home without needing a biopsy anymore and with complete resolution of symptoms and improvement of liver enzymes and AKI; AST 72, ALT 208, alkaline phosphatase 150, total bilirubin 0.5 and creatinine 0.5. She remained symptom-free with complete normalization of creatinine and liver enzymes at outpatient follow-up two weeks after discharge. She was eventually delivered of a healthy baby boy via cesarean section at 38 weeks of gestation with no peripartum complications.

## 3. Discussion

Differential diagnosis of nonacetaminophen fulminant hepatic failure includes hepatitis A and B, Epstein-Barr virus, and cytomegalovirus. In addition to the aforementioned etiologies, pregnant women especially in the second and third trimester are at higher risk for hemolysis, elevated liver enzymes and low platelets (HELLP) syndrome, Hepatitis E, and HSV. Herpes simplex virus, especially HSV2, is a common cause of mucocutaneous lesions, with prevalence ranges 10% to 60% in the general population [1]. HSV2 as a causative agent for hepatitis is a rare but serious complication that represents <2% of viral hepatitis and 0.8% of cases of acute liver failure, immunosuppression, and pregnancy during the third trimester are common precipitating factors [2]. Herpes simplex virus-2 (HSV-2) is a double-stranded DNA virus that belongs to Herpesviridae family of viruses, which includes other members such as herpes simplex virus-1 (HSV-1), varicella zoster virus, cytomegalovirus, Epstein-Barr virus, and human herpes viruses 6, 7, and 8 (Kaposi's sarcoma-associated herpesvirus) [3].

Potential mechanisms of herpes induced hepatitis include (i) a large HSV inoculum overwhelming the immune system on initial encounter; (ii) dissemination of mucosal herpetic lesions within a suppressed immune system; (iii) reactivation of a latent HSV reactivation or reinfection with a different new strain of HSV 4 (Hepatovirulent HSV) [4]. Disseminated HSV-2 infection has been defined by presence any of the following criteria, (a) positive HSV-2 IgM antibody test results, (b) HSV-2 DNA detected by polymerase chain reaction (PCR) assay of the peripheral blood mononuclear cells (PBSCs), or (c) involvement of two or more noncontiguous sites concurrently [5].

HSV hepatitis is one of the few treatable causes of acute liver failure when identified and treated promptly. When left untreated however, it is associated with high mortality, exceeding 80%–90% [6]. Consequently, it is been reasonable to start empiric treatment with acyclovir in patients with ALF of indeterminate etiology while awaiting confirmatory results, especially patients at risk for HSV. The high mortality rate of severe HSV hepatitis is a result of being difficult to diagnose in 77% of cases. The clinical manifestations of HSV hepatitis are not specific especially in the absence of typical mucocutaneous eruption that is only present in 50% of the cases. HSV hepatitis associated clinical findings that have been reported are fever (98%), clotting abnormality (96.5%), encephalitis (80%), and acute renal injury (65.3%) [7]. 90% of cases have low platelet count, low white blood cell count, and elevated transaminases (about 100‐ to 1000‐fold above normal with AST predominance) with normal to low bilirubin, thus named “anicteric hepatitis” like in our case [2]. The presence of these findings and patterns in a high-risk patient may support a diagnosis of HSV hepatitis, but their absence does not rule out a diagnosis. Poor prognostic parameters that might warrant the need for liver transplant evaluation are age > 40-years, male gender, coagulation abnormalities, the presence of an immunosuppressed state, encephalopathy, ALT > 5,000 U/L, platelet count < 75,000 U, and unavailability of acyclovir therapy [8].

It takes average of 2 to 3 weeks for the body to produce antibodies against HSV during which viral serology will be negative. HSV Immunoglobulin M (IgM) antibodies titer increases up to four times the normal value 2–4 weeks after the infection using enzyme-linked immunosorbent assay (ELISA) [9]. HerpeSelect HSV-2 ELISAs are glycoprotein G-based, type-specific antibody detection tests which are approved by the US Food and Drug Administration for diagnosis of genital herpes. Median interval from onset of symptoms to seroconversion is 21 days [10]. HSV PCR with concurrent elevation in aminotransferases like in our case is diagnostic and substitutes the need for invasive gold standard liver biopsy [2].

Treatment of HSV2 induced acute liver failure includes (i) supportive measures, (ii) addressing the underlying precipitating condition, (iii) antiviral agents, (vi) liver transplantation, and (v) in one report therapeutic plasma exchange [11]. The antiviral agent of choice is acyclovir. It is a safe drug and generally very well tolerated. Side effects if given orally are gastrointestinal upset, headache, and rash, while it can cause reversible nephrotoxicity if administered intravenously via formation of crystals and obstructive uropathy. Risk factors for nephrotoxicity are rapid intravenous administration, poorly hydrated patients, and those with preexisting renal disease who retain higher serum levels [12].

## 4. Conclusion

We emphasize maintaining a strong clinical index of suspicion for HSV2 induced hepatitis especially in the high-risk population even in the absence of suggestive serologies. This will help facilitate appropriate and prompt management. Given the safety profile of acyclovir and the high mortality rates of patients with HSV-related ALF, our case illustrates the need for empiric therapy in high-risk patient with otherwise unexplained hepatitis.

## References

[1] Gupta R., Warren T., Wald A. (2007). Genital herpes. *The Lancet*.

[2] Natu A., Iuppa G., Packer C. D. (2017). Herpes simplex virus hepatitis: a presentation of multi-institutional cases to promote early diagnosis and management of the disease. *Case Reports in Hepatology*.

[3] Fatahzadeh M., Schwartz R. A. (2007). Human herpes simplex virus infections: epidemiology, pathogenesis, symptomatology, diagnosis, and management. *Journal of the American Academy of Dermatology*.

[4] Yunce M., Bhat P., Jaganathan D., Bahrain M. (2019). Herpes hepatitis as a complication of total abdominal hysterectomy; an unusual complication of abdominal instrumentation. *Clinical Case Reports*.

[5] Kurosawa S., Sekiya N., Fukushima K. (2019). Unusual manifestation of disseminated herpes simplex virus type 2 infection associated with pharyngotonsilitis, esophagitis, and hemophagocytic lymphohisitocytosis without genital involvement. *BMC Infectious Diseases*.

[6] Jeong B. J., Tae H. J., Cho Y. J. (2016). Herpes simplex virus hepatitis treated with acyclovir. *The Ewha Medical Journal*.

[7] Norvell J. P., Blei A. T., Jovanovic B. D., Levitsky J. (2007). Herpes simplex virus hepatitis: an analysis of the published literature and institutional cases. *Liver Transplantation*.

[8] Masadeh M., Shen H., Lee Y. (2017). A fatal case of herpes simplex virus hepatitis in a pregnant patient. *Intractable and Rare Diseases Research*.

[9] Hook E. W., Cannon R. O., Nahmias A. J. (1992). Herpes simplex virus infection as a risk factor for human immunodeficiency virus infection in heterosexuals. *The Journal of Infectious Diseases*.

[10] Ashley-Morrow R., Krantz E., Wald A. (2003). Time course of seroconversion by Herpeselect ELISA after acquisition of genital Herpes simplex virus type 1 (HSV-1) or HSV-2. *Sexually Transmitted Diseases*.

[11] Holt E. W., Guy J., Gordon S. M. (2013). Acute liver failure caused by herpes simplex virus in a pregnant patient: Is there a potential role for therapeutic plasma exchange?. *Journal of Clinical Apheresis*.

[12] Arvin A., Campadelli-Fiume G., Mocarski E. (2019). *Human Herpesviruses: Biology, Therapy, and Immunoprophylaxis*.

